# The relationship of the transmural extent of T2-edema compared with the transmural extent of infarction: implications for the assessment of the area-at-risk

**DOI:** 10.1186/1532-429X-13-S1-O67

**Published:** 2011-02-02

**Authors:** Han W Kim, Ben Wince, Lowie van Assche, Wolfgang G Rehwald, Lubna B Bhatti, Deneen M Spatz, Igor Klem, Raymond J Kim

**Affiliations:** 1 Duke University, Durham, NC, USA

## Objective

To compare the transmural extent of hyperintensity (“edema”) on T2-weighted (T2W) CMR to the transmural extent of infarction by delayed-enhancement(DE)-CMR.

## Background

It has been reported that hyperintensity on T2W-CMR represents the area-at-risk. This conclusion is based upon the observation that the region of T2-hyperintensity is always larger than region of infarction. However, differences in measured size may be due to many factors, including the choice of different image intensity thresholds (Nat Rev Cardiol. 2010 Oct;7(10):547-9.). Additionally, basic physiology studies have established that the area-at-risk is not simply larger than the region of infarction, but is nearly always transmural regardless of the transmurality of infarct size. The relationship between the transmural extent of T2-hyperintensity and the transmural extent of infarction is unknown.

## Methods

34 canines underwent coronary occlusion of the LAD or the LCx followed by reperfusion. Occlusion time was varied between 45-120 minutes to produce a variable TEI. T2W-TSE and DE-CMR were performed at median of 3 days post MI. A standard T2W-TSE pulse sequence was used with vender supplied coil normalization (TE 80 ms, slice thickness 7mm, no gap) to obtain short-axis slices of the LV. T2W-TSE and DE-CMR images were separately scored on a per-infarct basis (blinded to the other technique) for the presence/absence of transmural T2-hyperintensity or transmural hyperenhancement, respectively, at any location within the heart. Additionally, the T2W and DE images were compared using a 6-point scale (0=0%, 1=1-25%, 2=26-50%, 3=51-75%, 4=76-99%, 5=100%) to determine the relative transmural extents.

## Results

**Figure 1:**
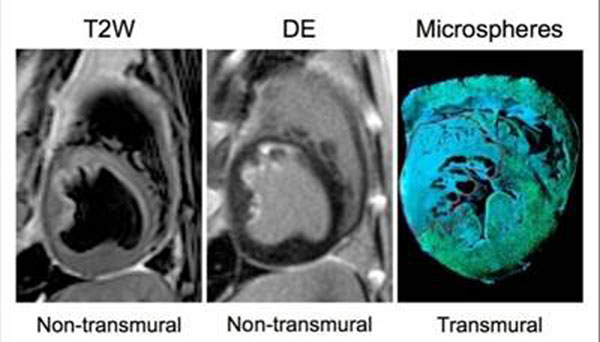


On a per-infarct basis, 59% (20/34) subjects were scored to have transmural T2-hyperintensity, which was similar to the proportion with transmural hyperenhancement (56%, 19/34). On a per-slice basis (n=244), transmural T2-hyperintensity at any location was found in 30.3% of slices, which was similar to the proportion of transmural hyperenhancement (32.0%, p=0.53. Table 1). Similarly, there was no difference in mean transmural extent score between DE (2.21.7) and T2W-CMR (2.21.7, p=0.38). Representative images from a subendocardial infarct are shown in Figure 1.

**Table 1:** 

Edema Transmural	HE Transmural	
	No	Yes

No	153	17
Yes	13	61

## Conclusion

T2-hyperintensity is frequently not transmural and is highly associated with the transmural extent of infarction. This is problematic for the use of T2W-TSE as a representation of the area-at-risk.

